# Exercise as a potent antidepressant intervention in Parkinson’s disease: a systematic review and meta-analysis of randomised controlled trials

**DOI:** 10.3389/fpsyg.2026.1846067

**Published:** 2026-05-14

**Authors:** Chu Sun, Yu Yan, Mengjing Zhu, Hui Ma, Huisong Xie

**Affiliations:** 1 College of Education, Beijing Sport University, Beijing, China; 2 Shenzhen Futian Innovation Middle School Affiliated to Shenzhen University, Shenzhen, China; 3 The School of International Education and Exchange, Beijing Sport University, Beijing, China

**Keywords:** depression, dose–response relationship, exercise, meta-analysis, Parkinson’s disease

## Abstract

**Background:**

Among the non-motor symptoms of Parkinson’s disease, depression ranks as one of the most common and debilitating, affecting roughly 40% of patients and substantially diminishing quality of life. Pharmacological treatments are often limited by side effects and variable efficacy, highlighting the need for effective non-pharmacological interventions. Although exercise has been proposed as a promising strategy, the optimal exercise prescription parameters for alleviating depressive symptoms in this population remain unclear.

**Methods:**

Five databases were searched from inception to December 15, 2025. Randomised controlled trials comparing exercise with non-exercise control groups in adults with Parkinson’s disease were included, with the primary outcome being change in depressive symptoms.

**Results:**

Twenty-one studies with 24 intervention arms comprising 1,010 participants were included. Exercise significantly reduced depressive symptoms compared with control groups (SMD = −0.93; *p* < 0.00001). Subgroup analyses showed that multicomponent exercise yielded the largest effect size (SMD = −1.22; *p* < 0.00001), followed by resistance exercise and aerobic exercise. Favorable parameters included session durations ≥ 60 min (SMD = −1.03; *p* < 0.00001), intervention durations > 8 weeks (SMD = −1.07; *p* < 0.00001), session frequencies < 3 times per week (SMD = −1.23; *p* < 0.00001), and total weekly exercise time < 180 min (SMD = −1.03; *p* < 0.00001). The overall certainty of evidence was moderate according to GRADE assessment.

**Conclusion:**

Exercise significantly alleviates depressive symptoms in people with Parkinson’s disease. For people with mild to moderate Parkinson’s disease with depression, multicomponent exercise appeared to be the most effective intervention type among those examined; however, aerobic and resistance exercise also significantly improved depressive symptoms. Favorable dosing was observed for sessions of 60 min or longer, program duration exceeding 8 weeks, and a frequency of less than three times per week with total weekly time under 180 min. Importantly, lower doses—including sessions shorter than 60 min, program duration of 8 weeks or less, frequency of three or more times per week, and total weekly time of 180 min or more—also produced significant antidepressant effects, demonstrating the flexibility of exercise prescription for this population. These findings support the incorporation of structured exercise programs into standard care for Parkinson’s disease with depressive symptomatology. Clinicians may consider these evidence-based parameters when prescribing exercise to this patient population while recognizing that multiple effective dosing options exist.

**Systematic review registration:**

Identifier CRD 420261350303.

## 1 Introduction

Ranking second only to Alzheimer’s disease, Parkinson’s disease (PD) is a prevalent neurodegenerative condition defined by progressive motor impairments such as bradykinesia, rigidity, and resting tremor (Hayes, 2019; Mehanna and Jankovic, 2019). Beyond these cardinal motor symptoms, there is a growing recognition of the profound burden imposed by non-motor symptoms, among which depression is one of the most prevalent and disabling (Schneider et al., 2017; Sveinbjornsdottir, 2016). Affecting approximately 40% of individuals with PD, depression significantly contributes to a reduced quality of life, accelerated cognitive decline, and increased caregiver distress (Agliardi and Guerini, 2025). Despite its high prevalence, depression in PD is often underdiagnosed and inadequately treated, with pharmacological interventions frequently limited by side effects, drug interactions, and variable efficacy (Lenka et al., 2017; Tarakad, 2025). This therapeutic gap underscores an urgent need for safe, accessible, and effective adjunctive interventions.

In recent years, increasing evidence supports exercise as a promising non-pharmacological strategy to target both motor and non-motor symptoms in PD (Padilha et al., 2023; Langeskov-Christensen et al., 2024; Mak and Wong-Yu, 2019). In this review, exercise is defined as planned, structured, repetitive physical activity undertaken with the goal of improving or maintaining physical fitness, health, or function, consistent with established definitions (Caspersen et al., 1985). Numerous randomised controlled trials (RCTs) have demonstrated the benefits of various exercise modalities—including aerobic training, resistance training, and mind–body exercises such as tai chi and yoga—on motor function, balance, and neuroplasticity (Bonavita, 2020; Schootemeijer et al., 2020). The rationale for exercise as a treatment for depression in PD is supported by converging lines of evidence, including its capacity to modulate monoaminergic neurotransmission, reduce systemic inflammation, and upregulate neurotrophic factors such as brain-derived neurotrophic factor (BDNF) (Han et al., 2025; Li et al., 2025; Hortobágyi et al., 2021). Moreover, exercise offers a low-risk, self-empowering approach that can be readily integrated into routine clinical care, positioning it as a potentially valuable intervention for depressive symptoms in this population.

Several previous meta-analyses have attempted to synthesise the evidence regarding exercise for depression in PD. While these studies have provided important foundational insights, they have been constrained by several limitations. For example, Tian et al. (2022) included a broad range of study designs and did not focus exclusively on RCTs, thereby limiting the strength of causal inferences. Similarly, Feller et al. (2023) pooled data across heterogeneous populations, and Omar Ahmad et al. (2023) employed non-specific depression outcome measures, introducing potential bias and imprecision in effect estimates. Furthermore, few studies, with the exception of Yan and Li (2025) have systematically examined the moderating effects of exercise type, intervention duration, or baseline depression severity, leaving critical questions unanswered regarding optimal prescription parameters. Consequently, the precise magnitude of the antidepressant effect of exercise in PD, and the factors that influence it, remain insufficiently characterized. By addressing the limitations of prior reviews and focusing exclusively on high-quality RCTs, this study seeks to provide robust evidence to inform clinical practice and guide the development of targeted exercise-based approaches for managing depression in this population.

## 2 Materials and methods

### 2.1 Design

Following the PRISMA 2020 guidelines, this study was conducted and registered in PROSPERO (registration number CRD420261350303).

### 2.2 Search strategy

Five electronic databases—PubMed, Web of Science, Cochrane Library, Scopus, and Embase—were systematically searched from their inception through December 15, 2025, using a combination of Medical Subject Headings and free-text keywords targeting Parkinson’s disease, exercise, and depression. The exact search terms used for PubMed were as follows: (“Parkinson Disease”[Mesh] OR “Parkinsonian Disorders”[Mesh] OR parkinson*[tiab] OR “parkinson’s disease”[tiab] OR “idiopathic parkinson*”[tiab] OR “paralysis agitans”[tiab]) AND (“Exercise”[Mesh] OR “Exercise Therapy”[Mesh] OR “Physical Therapy Modalities”[Mesh] OR “Resistance Training”[Mesh] OR “Physical Conditioning, Human”[Mesh] OR “Sports”[Mesh] OR “Walking”[Mesh] OR “Running”[Mesh] OR “Yoga”[Mesh] OR “Tai Ji”[Mesh] OR “Dance Therapy”[Mesh] OR “Qigong”[Mesh] OR “Physical Fitness”[Mesh] OR exercise*[tiab] OR “physical activity”[tiab] OR “physical training”[tiab] OR “physical therapy”[tiab] OR “physical exercise”[tiab] OR “aerobic exercise”[tiab] OR “aerobic training”[tiab] OR “resistance training”[tiab] OR “strength training”[tiab] OR “endurance training”[tiab] OR “balance training”[tiab] OR “gait training”[tiab] OR dance[tiab] OR dancing[tiab] OR yoga[tiab] OR “tai chi”[tiab] OR “tai ji”[tiab] OR qigong[tiab] OR pilates[tiab] OR walking[tiab] OR running[tiab] OR cycling[tiab] OR treadmill[tiab] OR “motor activity”[tiab]) AND (“Depression”[Mesh] OR “Depressive Disorder”[Mesh] OR “Mood Disorders”[Mesh] OR “Affective Symptoms”[Mesh] OR depression[tiab] OR “depressive disorder”[tiab] OR “depressive symptom*”[tiab] OR depressed[tiab] OR “mood disorder*”[tiab] OR “affective symptom*”[tiab] OR “major depressive disorder”[tiab] OR mdd[tiab] OR “emotional disorder*”[tiab]). Reference lists of relevant reviews were additionally scanned to capture any studies not identified electronically. The search and screening processes were handled independently by two reviewers, with any differences settled by consulting a third reviewer until unanimity was reached.

### 2.3 Eligibility criteria

Inclusion criteria comprise: (1) randomised controlled trial design; (2) an exercise intervention group and a non-exercise control group, where the non-exercise control group could include usual care, waiting-list, no intervention, or non-exercise active control such as health education or social activities, while studies comparing two different exercise modalities without a non-exercise control group were excluded; (3) adults diagnosed with Parkinson’s disease based on validated clinical criteria; (4) when medication was used, the dosage was required to be comparable between the exercise and control groups (i.e., no significant baseline differences or stable doses maintained equally across groups); and (5) depression assessed as a primary or secondary outcome using validated instruments.

Exclusion criteria included: (1) studies not published in English; (2) review articles or conference abstracts; (3) animal studies; (4) publications with a high risk of bias or without available full texts; and (5) studies lacking outcome data that could be quantified as mean and standard deviation.

### 2.4 Data extraction

The process of data extraction was conducted independently by two authors. When disagreements arose, they were resolved by referring to a third author. For each trial that met the inclusion criteria, the following information was collected: (1) general trial details, including the first author, publication year, country, and total number of participants; (2) intervention-related information, such as the mode of exercise, program duration, frequency per week, length of each session, weekly exercise volume, intensity, and who delivered the intervention; (3) participant background information, covering age and body mass index; and (4) outcome data, focusing specifically on changes in depression scores measured from before to after the intervention.

### 2.5 Methodological quality assessment

This study used the Cochrane Risk of Bias 2.0 tool to evaluate the quality of the included randomised controlled trials. Literature quality scoring was conducted independently by two authors. If any disagreement arose, a third author was consulted until consensus was reached.

### 2.6 Certainty of evidence

To assess the certainty of the primary outcome evidence, the GRADE (Grading of Recommendations Assessment, Development and Evaluation) system was utilized. Evidence quality was categorized into one of four levels—high, moderate, low, or very low—according to factors including risk of bias, inconsistency, indirectness, imprecision, and publication bias. The evaluation was carried out independently by two reviewers, with any disagreements settled through discussion until consensus was reached.

### 2.7 Statistical analysis

Analyses used Review Manager (5.4) and Stata (17.0). The primary outcome was change in depressive symptoms (continuous data). Due to varied depression scales, effect size was standardized mean difference (SMD) with 95% CI; negative SMD favored exercise. Heterogeneity was assessed using *I^2^* statistics, with values of 25, 50, and 75% indicating low, moderate, and high heterogeneity, respectively. A random-effects model was used when *I^2^* ≥ 50%; otherwise fixed-effects. In the context of this meta-analysis, a moderator was defined as a study-level characteristic (e.g., exercise type, session duration, intervention duration, session frequency, total weekly exercise time) that could systematically influence the direction or magnitude of the effect of exercise on depressive symptoms. Examining these moderators allows us to identify optimal exercise prescription parameters and to explain potential between-study heterogeneity. Subgroup analyses (exercise type, duration) and sensitivity analyses (sequential omission of studies) were performed. Publication bias was examined via funnel plots and Egger’s test (*p* < 0.05 significant) when ≥ 10 studies were included. Egger’s test quantifies funnel plot asymmetry by regressing the standardized effect estimate against its precision. The intercept (bias) of this regression indicates asymmetry: an intercept significantly different from zero (*p* < 0.05) suggests the presence of publication bias or other small-study effects. The coefficient (slope) reflects the relationship between effect size and precision; a slope close to zero indicates symmetry. The standard error of the coefficient measures its precision, and the 95% confidence interval provides the range within which the true asymmetry parameter is likely to fall (Egger et al., 1997).

## 3 Results

### 3.1 Studies selection

**Figure 1:**
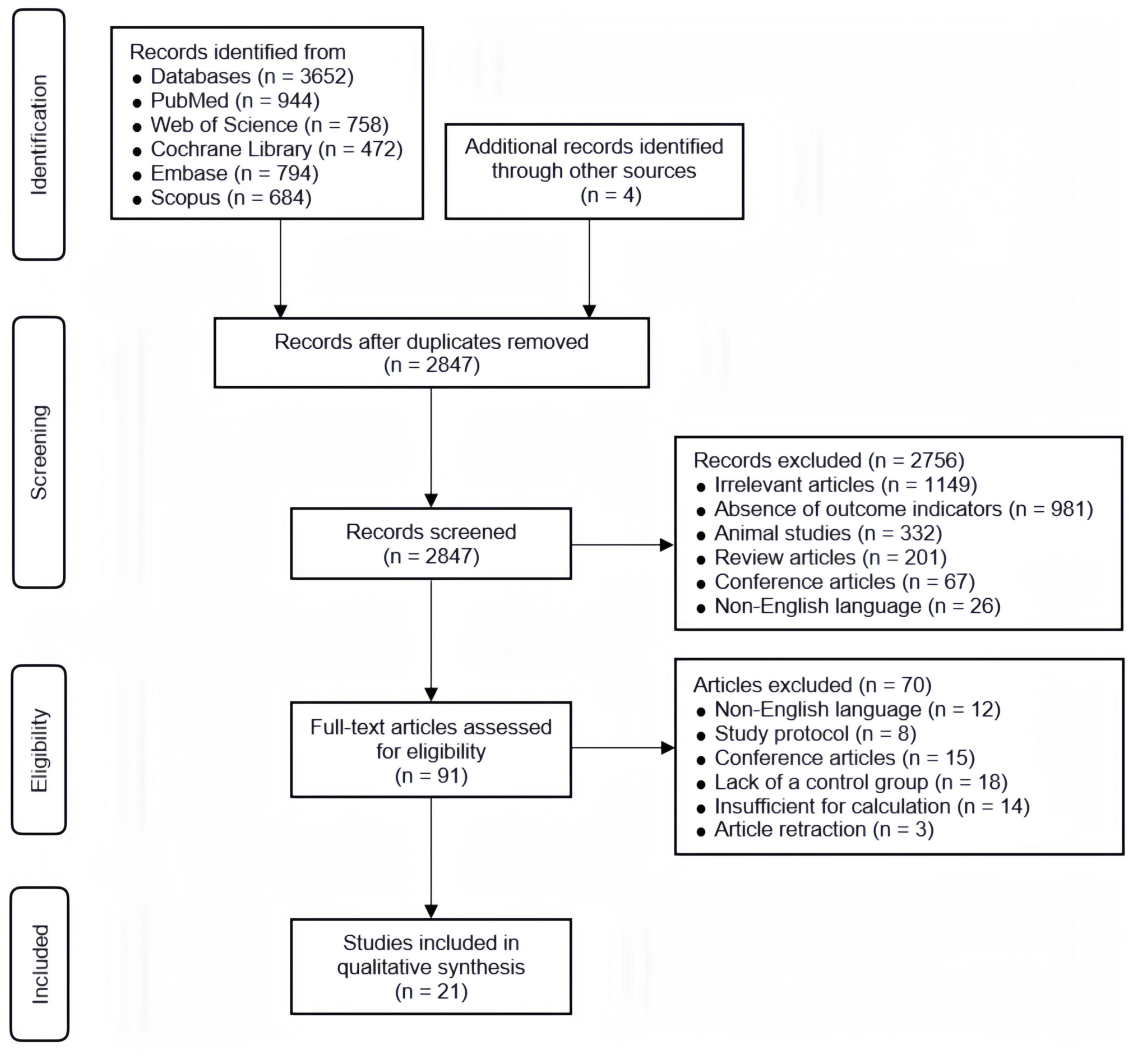
PRISMA flowchart of study selection.

Figure 1 presents the study selection process. A total of 3,652 records were retrieved from the five electronic databases, along with 4 additional records identified from other sources, such as reference lists of relevant reviews. After removing duplicates, 2,847 records underwent title and abstract screening, of which 2,756 were excluded for not meeting the eligibility criteria based on irrelevant topics or study design. The remaining 91 full-text articles were assessed in detail, leading to the exclusion of 70 articles. Ultimately, 21 studies (Hortobágyi et al., 2021; Duarte et al., 2023; Altmann et al., 2016; Dağ et al., 2022; Hashimoto et al., 2015; Kwok et al., 2019; Kwok et al., 2025; Lee et al., 2018; Lee et al., 2015; Cugusi et al., 2015; Li et al., 2022; Lima et al., 2019; Moon et al., 2020; Moratelli et al., 2025; Schmitz-Hübsch et al., 2006; Solla et al., 2019; Vitale et al., 2024; Tollár et al., 2018a,b; Wu et al., 2021; Tanaka et al., 2009) met the inclusion criteria and were included in this study.

### 3.2 Characteristics of the included studies

A total of 21 studies with 24 intervention arms met the inclusion criteria and were included in the systematic review and meta-analysis. The studies were published between 2006 (Schmitz-Hübsch et al., 2006) and 2025 (Moratelli et al., 2025), with the majority conducted in Europe, Asia, and South America. Specifically, four studies were conducted in China (Kwok et al., 2019; Kwok et al., 2025; Li et al., 2022; Wu et al., 2021), four in Brazil (Duarte et al., 2023; Lima et al., 2019; Moratelli et al., 2025; Tanaka et al., 2009), three in Italy (Cugusi et al., 2015; Solla et al., 2019; Vitale et al., 2024), two in Hungary (Tollár et al., 2018a,b), two in South Korea (Lee et al., 2018; Lee et al., 2015), and one each in the United States (Altmann et al., 2016), Germany (Schmitz-Hübsch et al., 2006), Japan (Hashimoto et al., 2015), the Netherlands (Hortobágyi et al., 2021), Turkey (Dağ et al., 2022), and a collaborative trial across multiple countries (Moon et al., 2020).

The total number of participants across all studies was 1,010, comprising 521 individuals in the exercise intervention groups and 489 in the control groups. Sample sizes varied widely, ranging from 17 (Moon et al., 2020) to 138 (Kwok et al., 2019) participants per trial. The mean age of participants ranged from 54.2 (Dağ et al., 2022) to 70.6 (Tollár et al., 2018a) years across studies. Most studies reported a balanced distribution of sex, with a slight predominance of male participants. Disease severity, as assessed by the Hoehn and Yahr scale, was predominantly classified as stage 1 to 3 (Duarte et al., 2023; Altmann et al., 2016; Dağ et al., 2022; Hashimoto et al., 2015; Kwok et al., 2019; Kwok et al., 2025; Lee et al., 2018; Cugusi et al., 2015; Li et al., 2022; Lima et al., 2019; Moon et al., 2020; Moratelli et al., 2025; Solla et al., 2019; Tollár et al., 2018b; Vitale et al., 2024), indicating mild to moderate disease in most participants (Hortobágyi et al., 2021; Tollár et al., 2018a,b). Few studies included patients with more advanced disease stages (Tollár et al., 2018a).

Regarding the exercise interventions, the included studies employed a variety of exercise modalities. Aerobic exercise (Duarte et al., 2023; Altmann et al., 2016; Dağ et al., 2022; Hashimoto et al., 2015; Lee et al., 2018; Cugusi et al., 2015; Solla et al., 2019; Vitale et al., 2024; Tollár et al., 2018a; Tanaka et al., 2009) was the most common type, including walking, nordic walking, arm crank training, and cycling, adopted in nine intervention arms. Multi-modal exercise (Hortobágyi et al., 2021; Hashimoto et al., 2015; Kwok et al., 2025; Lee et al., 2018; Li et al., 2022; Moon et al., 2020; Moratelli et al., 2025; Schmitz-Hübsch et al., 2006; Tollár et al., 2018a,b; Wu et al., 2021) programs, combining elements such as balance, strength, and flexibility training, were used in 10 intervention arms. Resistance training (Kwok et al., 2019; Lima et al., 2019) was applied in two intervention arms, and mind–body exercises, including yoga and qigong, were also represented. The duration of interventions ranged from 3 (Hashimoto et al., 2015; Tollár et al., 2018a) to 24 (Duarte et al., 2023) weeks, with session frequencies varying from once (Hashimoto et al., 2015; Kwok et al., 2019; Kwok et al., 2025) to five times (Lee et al., 2015; Li et al., 2022; Schmitz-Hübsch et al., 2006; Vitale et al., 2024) per week. Most interventions were supervised (Hortobágyi et al., 2021; Duarte et al., 2023; Altmann et al., 2016; Dağ et al., 2022; Hashimoto et al., 2015; Kwok et al., 2019; Kwok et al., 2025; Lee et al., 2018; Lee et al., 2015; Cugusi et al., 2015; Li et al., 2022; Lima et al., 2019; Moon et al., 2020; Moratelli et al., 2025; Schmitz-Hübsch et al., 2006; Solla et al., 2019; Vitale et al., 2024; Tollár et al., 2018a,b; Tanaka et al., 2009), with only one trial employing a home-based unsupervised program (Wu et al., 2021).

All studies included a non-exercise control group. Control conditions consisted of usual care, no intervention, or non-exercise active control such as health education or social activities. Depressive symptoms were assessed using validated instruments. The most frequently used scale was the Beck Depression Inventory (Hortobágyi et al., 2021; Altmann et al., 2016; Dağ et al., 2022; Lee et al., 2018; Lee et al., 2015; Cugusi et al., 2015; Moratelli et al., 2025; Solla et al., 2019; Vitale et al., 2024; Tollár et al., 2018a,b), employed in 12 studies, followed by the Hospital Anxiety and Depression Scale (Kwok et al., 2019; Kwok et al., 2025; Tanaka et al., 2009) in three studies, the Montgomery–Åsberg Depression Rating Scale (Duarte et al., 2023; Schmitz-Hübsch et al., 2006) in two studies, and the Self-rating Depression Scale (Hashimoto et al., 2015), Hamilton Depression Rating Scale (Li et al., 2022), and Geriatric Depression Scale (Moon et al., 2020) in one trial each. All outcome data were extracted as mean change scores with standard deviations from baseline to post-intervention, with missing data obtained through contact with corresponding authors when necessary.

Exercise intensity: Of the 21 included RCTs (with 24 intervention arms), 10 reported some form of intensity prescription. Aerobic exercise interventions commonly prescribed moderate intensity (e.g., 60–75% maximum heart rate, or Rate of Perceived Exertion 12–14). Resistance training protocols used 50–70% of one-repetition maximum or 8–12 repetitions to volitional fatigue. Multicomponent and mind–body interventions often described intensity qualitatively (e.g., “gentle,” “as tolerated”). Importantly, most studies allowed progressive adjustments over time, and intensity was not consistently measured or controlled across sessions, which precluded a quantitative subgroup analysis. The extracted intensity data are presented in Table 1.

**Table 1:** Characteristics of the studies included in this meta-analysis (Exercise dose characteristics).

Author/year	Intervention	Type of intervention	Frequency	Duration (weeks)	Minutes per session	Minutes per week	Intensity	Supervision	Supervisor
Altmann et al. (2016)	Running on a treadmill	Aerobic exercise	3	16	30	90	50–75% HRR	Yes	Fitness specialist certified in CPR and familiar with Parkinson’s disease
Cugusi et al. (2015)	Nordic Walking	Aerobic exercise	2	12	60	120	60–80% HRR	Yes	Adapted physical activity professionals
Dağ et al. (2022)	Arm crank ergometer training	Aerobic exercise	3	8	60	180	50–70% VO₂peak	Yes	Exercise specialist
Duarte et al. (2023)	Dance	Aerobic exercise	2	24	50	100	–	Yes	Research team
Hashimoto et al. (2015)	Dance	Aerobic exercise	1	12	60	60	50–70% HRR	Yes	Research team
Hashimoto et al. (2015)	Functional training	Multicomponent exercise	1	12	60	60	50–70% HRR	Yes	Research team
Hortobágyi et al. (2021)	Exergaming agility training	Multicomponent exercise	3	3	60	180	80% HRmax	Yes	Physical therapists
Kwok et al. (2019)	stretching and resistance training	Resistance exercise	1	8	90	90	–	Yes	Fitness instructor
Kwok et al. (2025)	Mindfulness yoga	Multicomponent exercise	1	8	90	90	–	Yes	Fitness instructor
Lee et al. (2015)	Virtual reality dance	Aerobic exercise	5	6	30	150	–	Yes	Experienced dance instructor
Lee et al. (2018)	Turo (Qi dance)	Multicomponent exercise	2	8	60	120	–	Yes	Experienced Qigong instructor
Li et al. (2022)	Health Qigong	Multicomponent exercise	5	12	60	300	–	Yes	Professional Qigong coach
Lima et al. (2019)	Resistance training	Resistance exercise	2	20	30	60	–	Yes	Exercise specialist
Moon et al. (2020)	Qigong	Multicomponent exercise	3	12	20	60	–	Yes	Research team
Moratelli et al. (2025)	Functional training and Mat Pilates	Multicomponent exercise	2	12	60	120	–	Yes	Physical education professionals trained in Pilates
Moratelli et al. (2025)	Functional training and mat Pilates	Multicomponent exercise	2	12	60	120	–	Yes	Physical education professionals trained in Pilate
Schmitz-Hübsch et al. (2006)	Qigong	Multicomponent exercise	1	16	90	90	–	Yes	Experienced Qigong teacher
Solla et al. (2019)	Sardinian folk dance	Aerobic exercise	2	12	90	180	–	Yes	Physical therapists
Tanaka et al. (2009)	Aerobic-oriented	Aerobic exercise	3	24	60	180	60–80% HRmax	Yes	Physical education professionals
Tollár et al. (2018b)	Multicomponent agility training	Multicomponent exercise	5	3	60	300	80% HRmax	Yes	Physical therapists
Tollár et al. (2018a)	Exergaming	Multicomponent exercise	5	5	60	300	–	Yes	Physical therapists
Tollár et al. (2018a)	Stationary cycling	Aerobic exercise	5	5	60	300	80% HRmax	Yes	Physical therapists
Vitale et al. (2024)	Biodanza	Aerobic exercise	1	12	120	120	80% HRmax	Yes	Physical therapists
Wu et al. (2021)	Home-based exercise (aerobic + resistance + stretching)	Multicomponent exercise	3	8	50	150	–	No	–

HRmax, maximum heart rate; HRR, heart rate reserve; VO₂peak, peak oxygen consumption, a measure of cardiorespiratory fitness.

As shown in Table 2, attrition rates were generally low across trials, indicating that loss to follow-up was well controlled and unlikely to bias the overall findings.

**Table 2:** Characteristics of the studies included in this meta-analysis (Participant characteristics).

Author/year	Country	Sample size (*n*)	Dropouts (*n*)	Reasons for dropouts	Male/female	Participants	Medication status	Mean age (y)	Depression outcomes
Altmann et al. (2016)	USA	Int:11 Con:10	4	Specific reasons not detailed	–	Hoehn & Yahr stages I–III	Stable antiparkinsonian and psychotropic medications; anticholinergics excluded	Int:62.8 ± 8.6 Con:67.8 ± 9.8	BDI-II
Cugusi et al. (2015)	Italy	Int:10 Con:10	0	–	Int:8/2 Con:8/2	Hoehn & Yahr stages I–III	All on dopaminergic therapy	Int:68.1 ± 8.7 Con:66.6 ± 7.3	BDI-II
Dağ et al. (2022)	Turkey	Int:13 Con:13	4	Failed to complete the training program	Int:9/4 Con:9/4	Hoehn & Yahr stages I–III	Stable medication	Int:57.23 ± 7.54 Con:58.23 ± 7.36	BDI-II
Duarte et al. (2023)	Brazil	Int:13 Con:13	5	–	Int:5/8 Con:5/8	Hoehn & Yahr stages I–III	Stable medication	Int:65.8 ± 5.1 Con:65.9 ± 6.5	MADRS
Hashimoto et al. (2015)	Japan	Int:15 Con:14	0	–	Int:3/12 Con:7/7	Hoehn & Yahr stages I–III	Stable medication	Int:67.9 ± 7.0 Con:69.7 ± 4.0	SDS
Hashimoto et al. (2015)	Japan	Int:17 Con:14	0	–	Int:2/15 Con:7/7	Hoehn & Yahr stages I–III	Stable medication	Int:62.7 ± 14.9 Con:69.7 ± 4.0	SDS
Hortobágyi et al. (2021)	Netherlands	Int:19 Con:26	6	Failed to complete the training program	Int:11/8 Con:15/11	Hoehn & Yahr stages II–III	Stable medication	Int:67.5 ± 3.9 Con:67.8 ± 3.8	BDI-II
Kwok et al. (2019)	China	Int:67 Con:71	26	Specific reasons not detailed	Int:28/39 Con:37/34	Hoehn & Yahr stages I–III	Patients on antidepressants excluded	Int:63.5 ± 9.3 Con:63.7 ± 8.2	HADS
Kwok et al. (2025)	China	Int:53 Con:54	24	Specific reasons not detailed	Int:28/25 Con:20/34	Hoehn & Yahr stages I–III	Antidepressants and benzodiazepines	Int:64.3 ± 8.0 Con:63.3 ± 7.5	HADS
Lee et al. (2015)	South Korea	Int:10 Con:10	0	–	Int:5/5 Con:5/5	–	Stable medication	Int:68.4 ± 2.9 Con:70.1 ± 3.3	BDI-II
Lee et al. (2018)	South Korea	Int:25 Con:16	6	Moved (1), lost contact (4), traffic accident death (1)	Int:10/15 Con:7/9	Hoehn & Yahr stages I–III	Maintained regular pharmacotherapy	Int:65.8 ± 7.2 Con:65.7 ± 6.4	BDI-II
Li et al. (2022)	China	Int:18 Con:18	6	Prior Qigong experience (1), incomplete (2), other exercise (1), incomplete data (1), did not complete (1)	Int:7/11 Con:8/10	Hoehn & Yahr stages I–III	No changes in medication	Int:66.33 ± 10.89 Con:69.17 ± 6.48	HDRS-17
Lima et al. (2019)	Brazil	Int:17 Con:16	0	–	–	Hoehn & Yahr stages I–III	Levodopa + Carbidopa Prolopa Benserazide Sifrol (pramipexole) Biperiden	Int: 66.2 ± 5.5 Con: 67.2 ± 5.2	HAM-D17
Moon et al. (2020)	USA	Int:8 Con:9	4	Lost interest,other reasons not reported	Int:4/4 Con:3/6	Hoehn & Yahr stages I–III	Stable medication	Int: 66.4 ± 8.1 Con: 65.9 ± 5.4	GDS
Moratelli et al. (2025)	Brazil	Int:12 Con:10	3	Lack of interest/availability, other diseases (PwP); not reported (healthy)	–	Hoehn & Yahr stages I–III	Stable medication	–	BDI-II
Moratelli et al. (2025)	Brazil	Int:13 Con:10	3	Lack of interest/availability, other diseases;	–	Hoehn & Yahr stages I–III	Anxiolytics/antidepressants; sleep medications	–	BDI-II
Schmitz-Hübsch et al. (2006)	Germany	Int:32 Con:24	7	–	Int:24/8 Con:19/5	Hoehn & Yahr stages I–IV	Stable medication	Int: 64 ± 8 Con: 63 ± 8	MADRS
Solla et al. (2019)	Italy	Int:10 Con:10	1	Excluded due to severe dyskinesia/freezing	Int:6/4 Con:7/3	Hoehn & Yahr stages I–III	Stable medication	Int: 67.8 ± 5.9 Con: 67.1 ± 6.3	BDI-II
Tanaka et al. (2009)	Brazil	Int:10 Con:10	0	–	–	Hoehn & Yahr stages I–III	Stable medication	Int:64.80 ± 8.49 Con:64.60 ± 6.25	HADS
Tollár et al. (2018b)	Hungary	Int:35 Con:20	–	–	Int:17/18 Con:12/8	Hoehn & Yahr stages II–III	Majority on levodopa or dopamine agonists	Int: 67.3 ± 3.4 Con: 67.6 ± 4.1	BDI-II
Tollár et al. (2018a)	Hungary	Int:25 Con:24	0	–	Int:12/13 Con:13/11	Hoehn & Yahr stages II–III	Stable medication	Int: 70.0 ± 4.7 Con: 67.5 ± 4.3	BDI-II
Tollár et al. (2018a)	Hungary	Int:25 Con:24	0	–	Int:11/14 Con:13/11	Hoehn & Yahr stages II–IV	Stable medication	Int: 70.6 ± 4.1 Con: 67.5 ± 4.3	BDI-II
Vitale et al. (2024)	Italy	Int:14 Con:14	0	–	Int:10/4 Con:12/2	Hoehn & Yahr stages I–III	Stable medication	Int: 64.6 ± 9.0 Con: 64.1 ± 6.6	BDI-II
Wu et al. (2021)	China	Int:49 Con:49	0	–	Int:26/23 Con:30/19	Hoehn & Yahr stages I–II	Stable medication	Int: 63.65 ± 6.02 Con: 66.59 ± 8.61	GDS-15

BDI-II, Beck Depression Inventory-II; MADRS, Montgomery-Åsberg Depression Rating Scale; SDS, Self-Rating Depression Scale; HADS, Hospital Anxiety and Depression Scale; HDRS-17/HAM-D17, Hamilton Depression Rating Scale; GDS/GDS-15, Geriatric Depression Scale; Int, intervention group; Con, control group; Hoehn & Yahr, used to classify the severity of Parkinson’s disease based on motor symptoms (stages I–V) (Hoehn and Yahr, 1967).

### 3.3 Main effect

**Figure 2:**
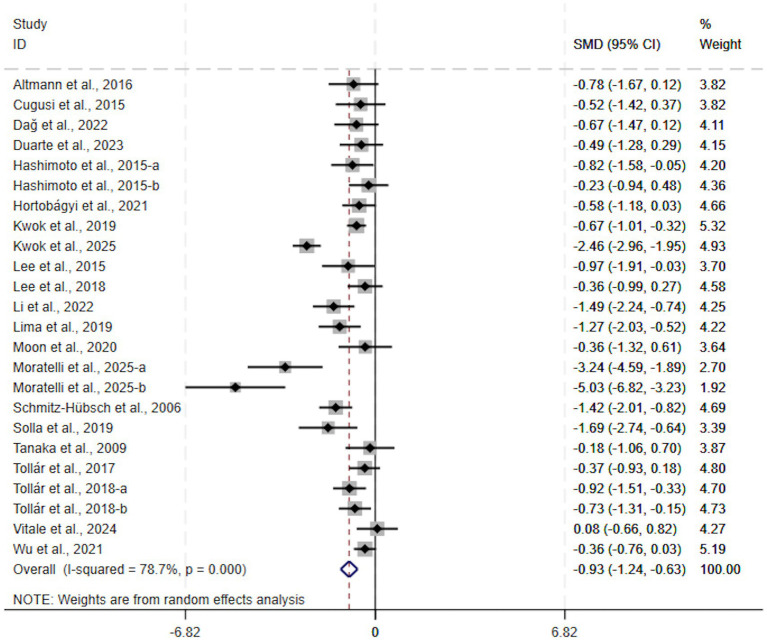
A meta-analysis of the effects of exercise on depression in people with Parkinson’s disease.

Relative to the control group, exercise produced a significant improvement in depressive symptoms among Parkinson’s disease patients (SMD, −0.93; 95% CI, −1.24 to −0.63, *p* < 0.00001, *I*^2^ = 79%, Figure 2). The meta-analysis revealed substantial heterogeneity across the included studies. With the aim of elucidating the basis of this inconsistency and recognizing adjustable components of exercise, extra analytical procedures (subgroup and sensitivity analyses) were executed.

### 3.4 Subgroup analysis

**Figure 3:**
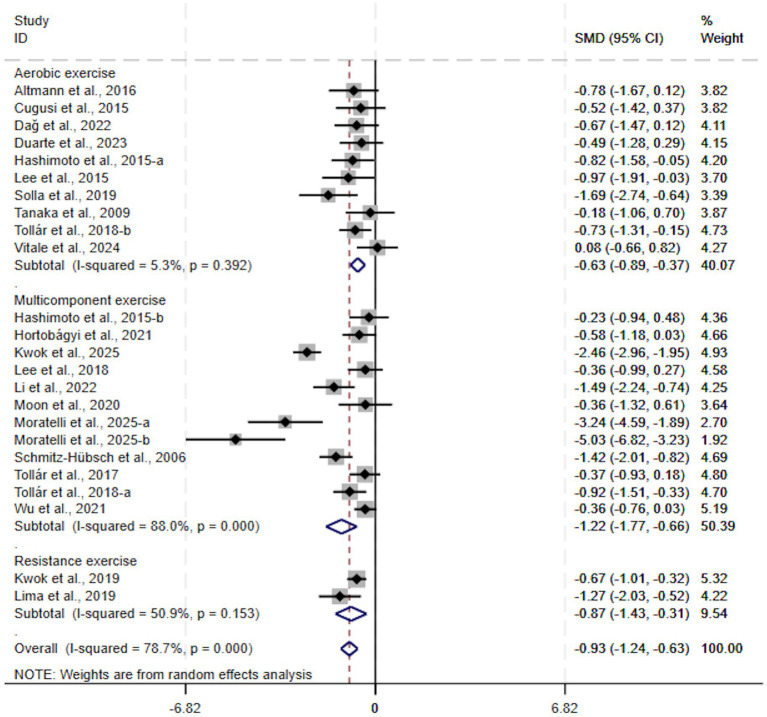
Subgroup analysis by intervention type.

When stratified by intervention type, aerobic exercise (SMD, −0.63; 95% CI, −0.89 to −0.37, *p* < 0.00001, *I*^2^ = 5%, Figure 3), resistance exercise (SMD, −0.87; 95% CI, −1.43 to −0.31, *p* = 0.002, *I*^2^ = 51%, Figure 3), and multicomponent exercise (SMD, −1.22; 95% CI, −1.77 to −0.66, *p* < 0.00001, *I*^2^ = 88%, Figure 3) all demonstrated significant therapeutic effects, with multicomponent exercise showing the largest effect size and the most pronounced efficacy.

**Figure 4:**
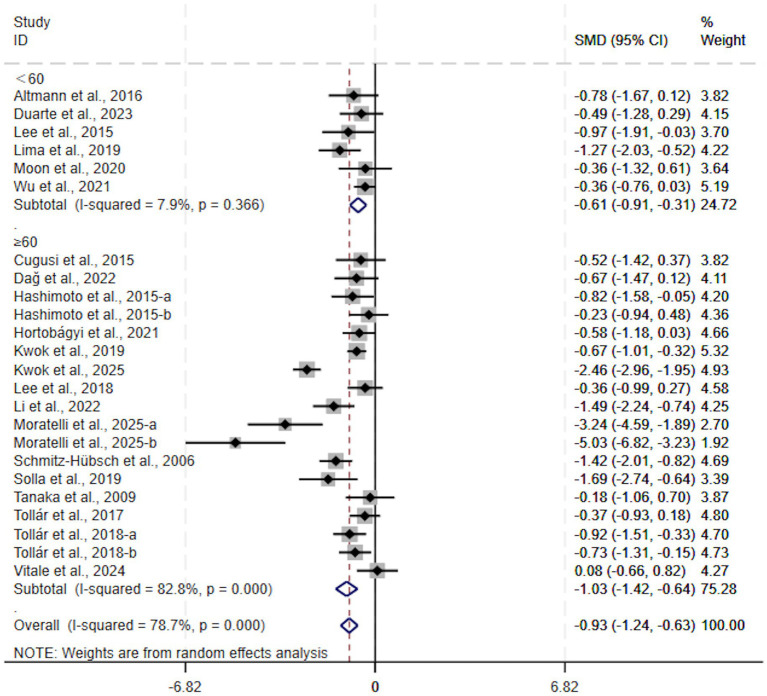
Subgroup analysis by session duration.

With regard to session duration, exercise interventions significantly alleviated depressive symptoms in patients with Parkinson’s disease irrespective of the length of each session. For sessions lasting 60 min or longer, the effect was significant (SMD, −1.03; 95% CI, −1.42 to −0.64, *p* < 0.00001, *I*^2^ = 83%, Figure 4). For sessions lasting less than 60 min, the effect was also significant (SMD, −0.61; 95% CI, −0.91 to −0.31, *p* < 0.0001, *I*^2^ = 8%, Figure 4). The improvement was more pronounced in the group with sessions of 60 min or longer.

**Figure 5:**
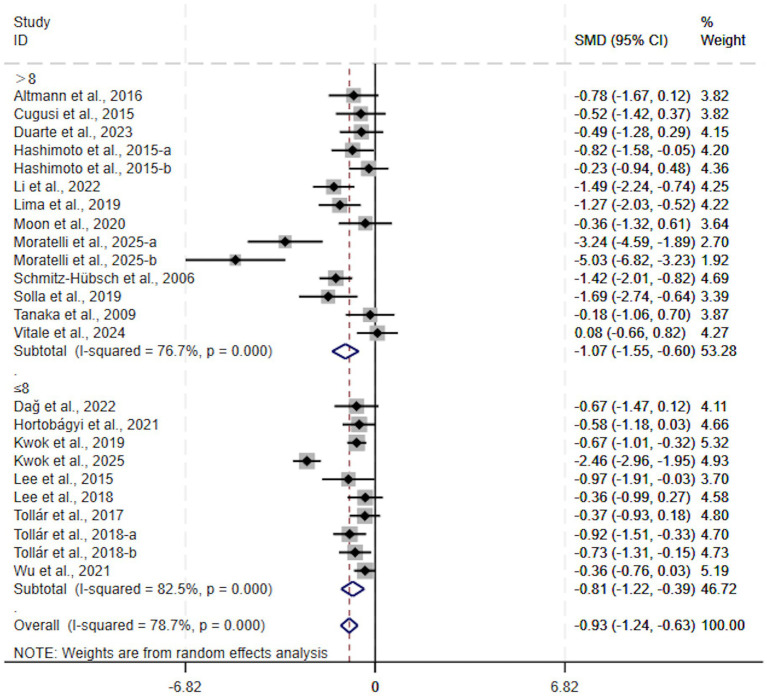
Subgroup analysis by intervention duration.

Concerning intervention duration, exercise interventions lasting > 8 weeks (SMD, −1.07; 95% CI, −1.55 to −0.60, *p* < 0.00001, *I*^2^ = 77%, Figure 5) and those lasting ≤ 8 weeks (SMD, −0.81; 95% CI, −1.22 to −0.39, *p* = 0.0001, *I*^2^ = 82%, Figure 5) significant improvements were observed in both depressive symptoms in patients with Parkinson’s disease. Both durations were effective, and the advantage was more evident in interventions lasting > 8 weeks.

**Figure 6:**
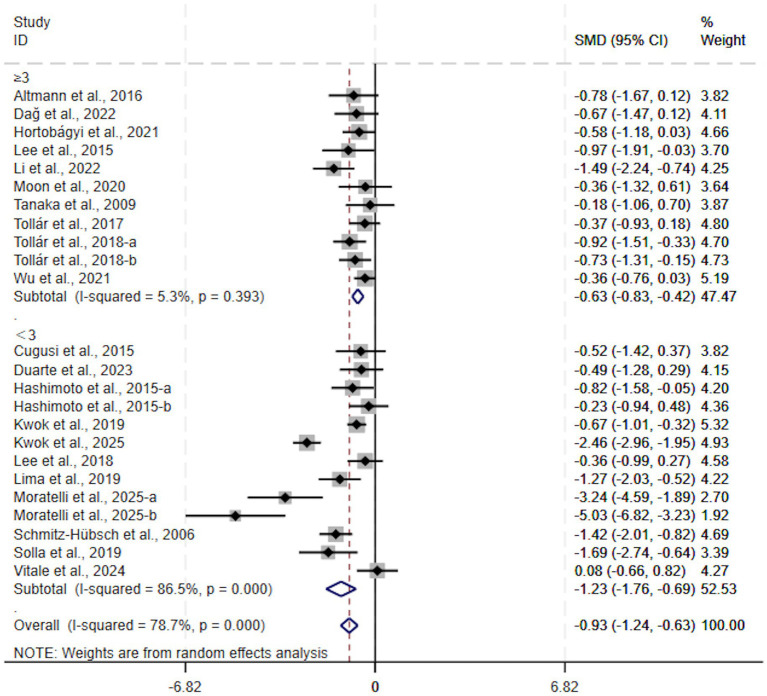
Subgroup analysis by session frequency.

For session frequency, both interventions conducted < 3 times per week (SMD, −1.23; 95% CI, −1.76 to −0.69, *p* < 0.00001, *I*^2^ = 86%, Figure 6) and those performed ≥ 3 times per week (SMD, −0.63; 95% CI, −0.83 to −0.42, *p* < 0.00001, *I*^2^ = 5%, Figure 6) significantly reduced depressive symptoms. Of note, a more pronounced treatment effect was observed in the group exercising < 3 times weekly.

**Figure 7:**
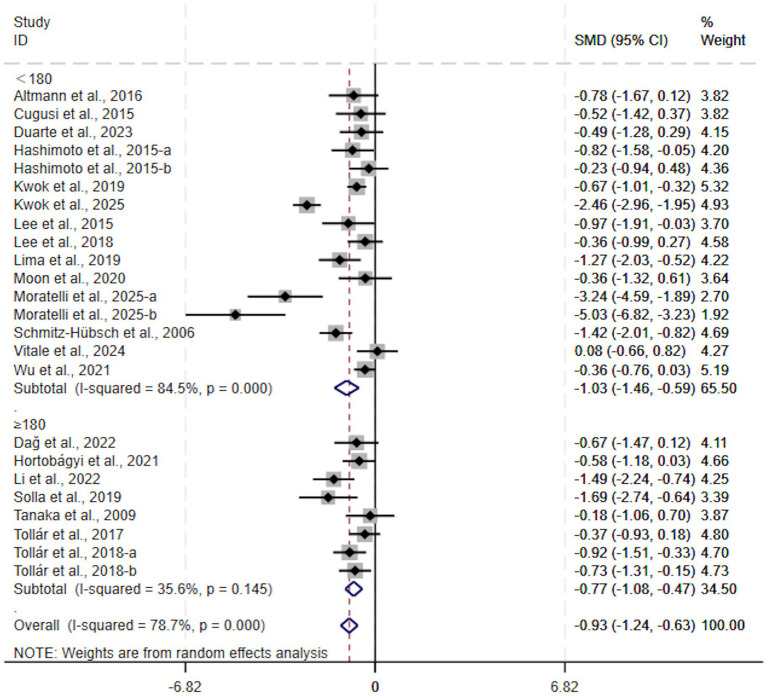
Subgroup analysis by total weekly exercise time.

Further analysis of total weekly exercise time revealed that both interventions with 180 min or more per week (SMD, −0.77; 95% CI, −1.08 to −0.47, *p* < 0.00001, *I*^2^ = 36%, Figure 7) and those with less than 180 min (SMD, −1.03; 95% CI, −1.46 to −0.59, *p* < 0.00001, *I*^2^ = 84%, Figure 7) per week significantly improved depressive symptoms in patients with Parkinson’s disease. Among these, interventions with less than 180 min of weekly exercise time showed superior efficacy.

### 3.5 Risk of bias

**Figure 8:**
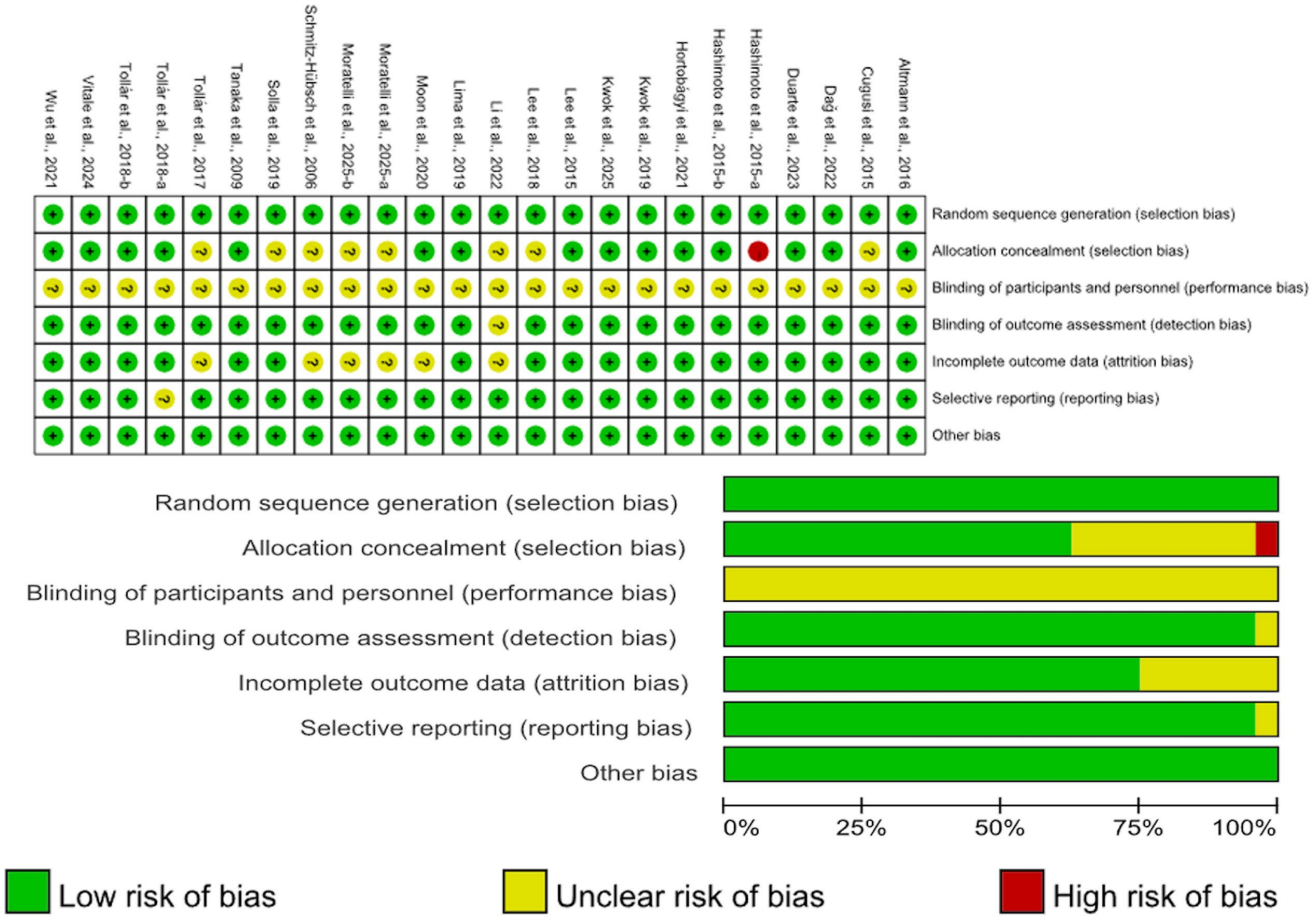
Risk assessment of included studies.

To assess methodological quality, the Rob-2.0 tool was applied to the 21 included randomised controlled trials (Figure 8). A well-documented limitation in exercise intervention research is the practical impossibility of blinding participants and exercise providers, which contributed to a high or unclear risk of performance bias in the majority of studies. In contrast, the risk of bias across other key domains was consistently low.

### 3.6 GRADE evaluation of evidence quality

Certainty in the primary outcome evidence was assessed using the GRADE framework, as summarized in Table 3. The overall evidence was rated as moderate. The quality was downgraded by one level due to substantial heterogeneity across studies (*I*^2^ = 79%), while no serious concerns were identified regarding risk of bias, indirectness, or imprecision.

**Table 3:** GRADE assessment of primary outcomes.

Outcome	No of participants (studies)	Certainty assessment					SMD (95% CI)	GRADE^*^
		Risk of bias	Inconsistency	Indirectness	Imprecision	Other considerations		
Depression	1,010 (24 RCTs)	Not serious	Serious	Not serious	Not serious	Not serious	−0.93 [−1.24, −0.63]	⊕ ⊕ ⊕◯ Moderate

The * indicates that the GRADE assessment presented is a modified or adapted version tailored to the specific context of this study.

### 3.7 Publication bias

**Figure 9:**
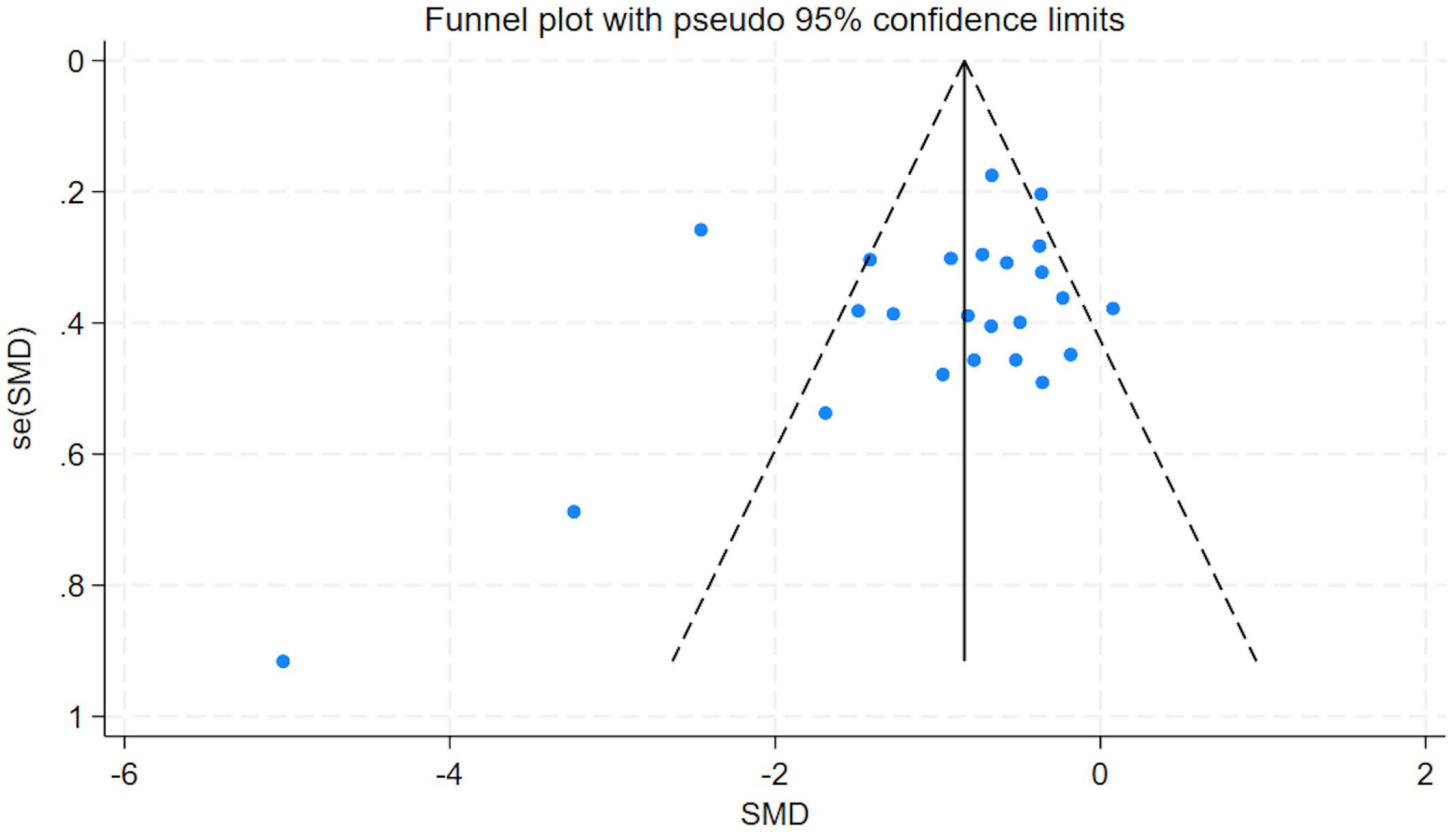
Results of funnel plot.

Publication bias was assessed using funnel plots and the Egger test. The funnel plots showed a symmetrical distribution (Figure 9), and the *p*-value from the Egger test was greater than 0.05 (*t* = −0.57, *p* = 0.573, Table 4), indicating that there was no statistically significant publication bias among the included studies. Additionally, a few small-sample studies fell outside the pseudo 95% confidence limits in the funnel plot, which likely reflects imprecision and between-study heterogeneity rather than definitive publication bias; this interpretation is supported by the nonsignificant Egger test and the stable sensitivity analyses.

**Table 4:** Results of Egger’s test (depression).

Std_Eff	Coefficient	Std. err.	*t*	*P* > |*t*|	[95% conf. interval]
Slope	−0.2485383	0.4339542	−0.57	0.573	(−1.148504, 0.6514276)
Bias	−1.863695	1.297503	−1.44	0.165	(−4.554552, 0.8271612)

Std. Err, standard error; *t*, *t*-test statistic; *p*, probability.

### 3.8 Sensitivity analyses

**Figure 10:**
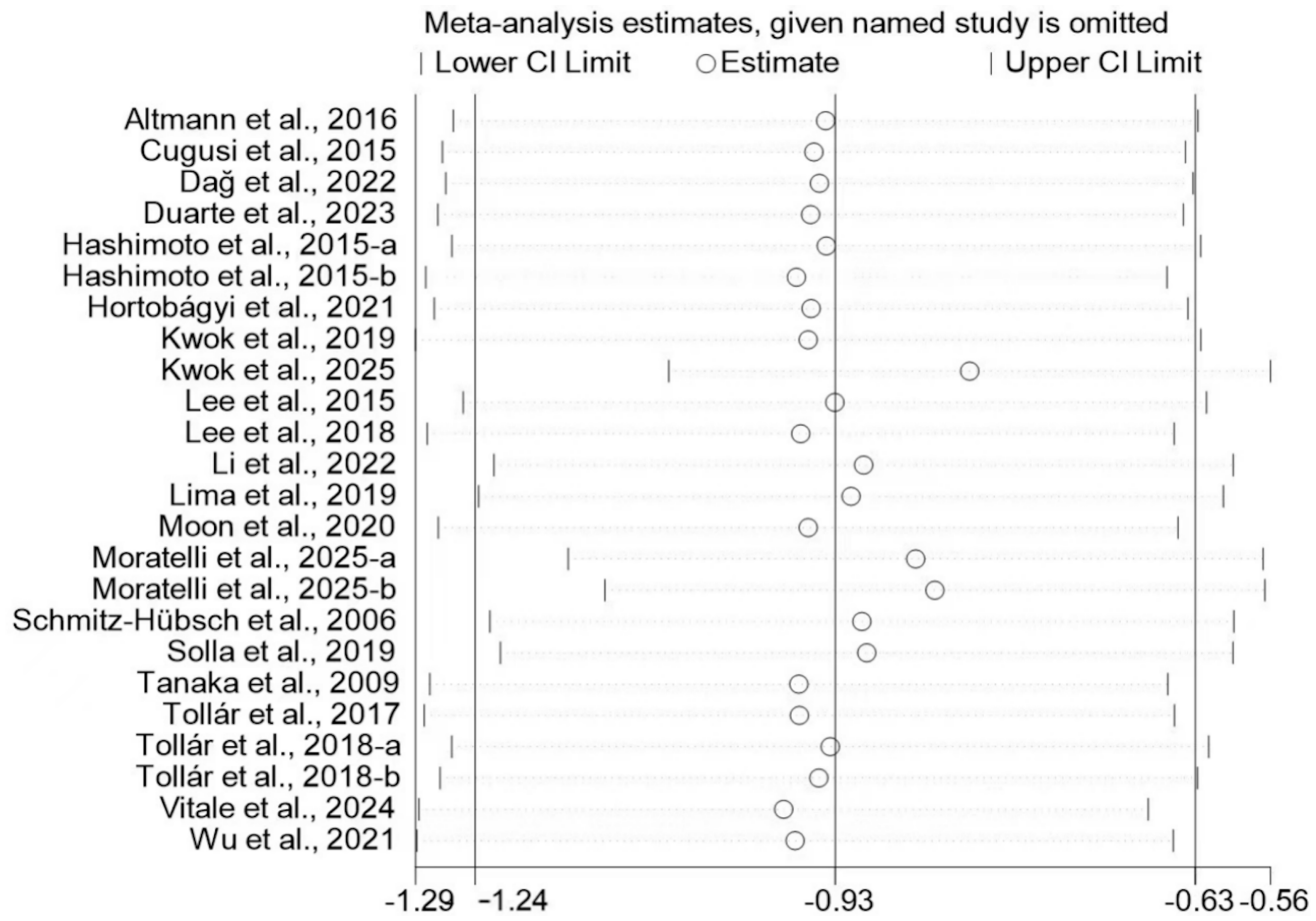
Results of sensitivity analyses.

Sensitivity analyses were performed by sequentially omitting each individual trial to evaluate the robustness of the pooled estimate, as shown in Figure 10. The results remained stable and consistent across all iterations, with no single trial exerting a disproportionate influence on the overall effect size.

## 4 Discussion

### 4.1 Main findings

This study aimed to evaluate the effect of exercise on depressive symptoms in patients with Parkinson’s disease and to explore favorable exercise parameters for this population. The results showed that exercise significantly alleviated depressive symptoms, with multicomponent exercise emerging as the most effective intervention type among those examined. Subgroup analyses further indicated that more pronounced antidepressant effects were associated with sessions lasting 60 min or longer, intervention durations exceeding 8 weeks, frequencies of less than three times per week, and total weekly exercise time under 180 min.

### 4.2 Effects of exercise on depressive symptoms

Recent meta-analyses have substantially advanced our understanding of exercise as an antidepressant intervention in Parkinson’s disease (PD). Gollan et al. (2022) specifically examined resistance training (RT), reporting that RT significantly improves depression in PD patients compared with passive controls, but is not superior to other physically active interventions, suggesting that exercise type may be of secondary interest. Costa et al. (2024) conducted a comprehensive meta-analysis, revealing a moderate-to-large effect for depression when comparing exercise with non-exercise controls; notably, mind–body exercises yielded the largest effect, followed by resistance and aerobic modalities. Ren et al. (2025) focused specifically on aerobic exercise, reporting a significant antidepressant effect and further identifying optimal prescription parameters. Tan et al. (2025) synthesized evidence on traditional Chinese exercises for neuropsychiatric symptoms, reporting substantial improvements in depression and anxiety, with interventions lasting up to 12 weeks showing more significant anti-depression effects. Li et al. (2025) employed a network meta-analysis comparing 25 exercise interventions, revealing that dance exercise was more effective than other modalities in reducing depression. Collectively, these prior meta-analyses reinforce the conclusion that multiple exercise modalities produce clinically meaningful antidepressant effects in PD—with mind–body, multicomponent, and dance-based interventions showing particularly promising results—and highlight the importance of individualized, evidence-based exercise prescription, providing a robust foundation for the present meta-analysis. Building on these findings, the present meta-analysis extends the key results of prior meta-analyses to specific exercise prescription parameters for individuals with mild to moderate Parkinson’s disease and depressive symptomatology, representing a first step toward clinical exercise prescription for this population.

The present findings demonstrate that exercise significantly alleviates depressive symptoms in patients with Parkinson’s disease, a result that is broadly consistent with prior research. For instance, a previous meta-analysis (Goodwin et al., 2008) reported that exercise interventions led to significant improvements in depressive symptoms among this population, with effect sizes ranging from moderate to large. Similarly, a systematic review (Song et al., 2017) focusing on non-pharmacological interventions for depression in Parkinson’s disease concluded that exercise consistently produced beneficial effects across diverse study settings. These converging lines of evidence reinforce the role of structured physical activity as a viable non-pharmacological strategy for managing depressive symptoms in Parkinson’s disease.

The antidepressant effects of exercise in Parkinson’s disease are likely mediated by a complex interplay of neurobiological, physiological, and psychological mechanisms (Zhang et al., 2021; van Wegen et al., 2024). At the molecular level, physical activity promotes the release of monoamines—including serotonin, dopamine, and norepinephrine—all of which are directly involved in mood regulation and are typically deficient in Parkinson’s disease (Khedr et al., 2007). These neurochemical changes mirror the mechanisms of action of conventional antidepressant medications (Khedr et al., 2007; Fifel and Videnovic, 2019). Moreover, exercise upregulates brain-derived neurotrophic factor and other neurotrophins, thereby supporting neuroplasticity, synaptic connectivity, and neuronal survival in mood-related brain regions such as the hippocampus and prefrontal cortex (Costa et al., 2012). These structural and functional adaptations may contribute to the restoration of impaired neural circuits associated with depression.

In addition to these neurochemical and neuroplastic effects, regular exercise exerts systemic anti-inflammatory effects by reducing levels of pro-inflammatory cytokines, which have been implicated in the pathophysiology of both Parkinson’s disease and major depressive disorder (Lo Buono et al., 2021). This immunomodulatory role may be particularly relevant, given growing evidence that neuroinflammation contributes to the progression of both motor and non-motor symptoms in Parkinson’s disease.

At the physiological level, exercise is known to enhance cardiovascular function and cerebral blood flow, which may improve oxygen and nutrient delivery to brain regions involved in affective regulation (Kim et al., 2025). Resistance training, in particular, helps counteract muscle atrophy and preserve physical function, changes that may indirectly support mental health by maintaining mobility, independence, and overall physical wellbeing (Koutsouras et al., 2020; Lauzé et al., 2016).

From a psychological perspective, engagement in regular exercise fosters improvements in self-efficacy and mental resilience, both of which are important buffers against depressive symptoms (Kwok et al., 2023). Physical activity also provides a sense of accomplishment and mastery, particularly when structured as goal-oriented training (Cruise et al., 2011). Furthermore, group-based exercise interventions offer opportunities for social interaction, which can effectively reduce feelings of isolation and helplessness—common experiences among individuals with Parkinson’s disease (Ernst et al., 2024). These psychosocial benefits may be especially meaningful in a population where social withdrawal often compounds the burden of depressive illness.

Despite the overall positive findings, not all studies have reported consistent effects. Some investigations have failed to detect statistically significant improvements in depressive symptoms following exercise interventions. These discrepancies may be attributable to several factors, including differences in intervention protocols (e.g., type, frequency, intensity, and duration of exercise), variability in baseline depression severity across trial populations, differences in control group conditions (e.g., active versus passive controls), and the use of diverse depression assessment instruments. Additionally, variations in sample size and statistical power across studies may have influenced the detectability of treatment effects.

Given the substantial heterogeneity in this meta-analysis, we conducted subgroup analyses to further clarify the link between specific exercise parameters and antidepressant efficacy. These analyses aimed to identify potentially favorable intervention characteristics, thereby offering more precise guidance for clinical prescription and future research design.

### 4.3 Effects of various exercise moderators on depressive symptoms

Subgroup analyses demonstrated that multicomponent exercise was the most effective intervention among the modalities assessed. Favorable outcomes for depressive symptoms in patients with Parkinson’s disease were associated with session durations of 60 min or longer, intervention periods exceeding 8 weeks, frequencies of fewer than three sessions per week, and total weekly exercise time under 180 min.

Our findings indicated that, among the various exercise modalities examined, multicomponent exercise was particularly effective in reducing depressive symptoms in patients with Parkinson’s disease. In contrast, while aerobic exercise and resistance exercise also demonstrated significant effects, the effect size for multicomponent exercise was substantially larger. Several factors may explain why multicomponent interventions appear particularly beneficial for this population. First, Parkinson’s disease is characterized by multifaceted impairments encompassing motor, cognitive, and affective domains (Leta et al., 2025; O’Callaghan and Lewis, 2017). Multicomponent exercise, which integrates elements such as balance, strength, flexibility, and agility training, may address these interconnected deficits more comprehensively than single-modality interventions (Yan et al., 2024; Angulo et al., 2020). The synergistic effects of combining different movement types may enhance neuroplasticity through diverse neural pathways, potentially leading to greater improvements in mood regulation (Ribas et al., 2017). Second, the social and cognitive engagement inherent in many multicomponent programs—such as dance-based interventions, functional training, or mind–body exercises like yoga and qigong—may provide additional psychological benefits beyond the physiological effects of exercise alone (Kwok et al., 2017). These activities often involve learning new movement sequences, group interaction, and mindful attention, which may contribute to reduced depressive symptoms through mechanisms such as increased self-efficacy, social connectedness, and cognitive stimulation (Hashimoto et al., 2015; Rios Romenets et al., 2015). A mechanistic review by Zikereya et al. (2023) noted that multicomponent exercise may restore goal-directed and habitual control circuits disrupted in PD, offering synergistic mood benefits.

Aerobic exercise also demonstrated significant efficacy in reducing depressive symptoms, albeit with a smaller effect size compared with multicomponent exercise. The consistent effects observed across aerobic exercise studies, reflected by low heterogeneity, support its reliability as an intervention option. Several factors may account for the relatively modest effect size. Aerobic interventions in the included studies varied considerably in terms of intensity, delivery format, and setting, which may have contributed to variability in outcomes. Moreover, while aerobic exercise robustly enhances cardiovascular fitness and systemic physiological pathways, the complex symptomatology of Parkinson’s disease—involving both motor and non-motor domains—may require more comprehensive stimulation to achieve optimal antidepressant effects (Moratelli et al., 2025; Ridgel et al., 2016). Nevertheless, the established benefits of aerobic exercise on cardiovascular health, motor function, and neuroplasticity make it a valuable component of exercise prescription for this population (Altmann et al., 2016; Moon et al., 2020).

In contrast, only two resistance exercise studies met the inclusion criteria, which limits the robustness of findings for this modality. The relative scarcity of resistance training studies in Parkinson’s disease may reflect practical considerations. Patients often present with motor symptoms such as bradykinesia, rigidity, and postural instability, which may pose challenges for performing traditional resistance exercises, particularly those requiring precise form or free-weight handling (Silva-Batista et al., 2017). Concerns about safety, fall risk, and the need for specialised equipment or supervision may have constrained the design and implementation of resistance exercise trials (Hirsch et al., 2003). Despite the limited number of studies, the observed effect size for resistance exercise was substantial and statistically significant, suggesting that this modality warrants further investigation.

Our study found that exercise interventions exceeding 8 weeks in duration were associated with significantly greater amelioration of depressive symptoms compared with shorter interventions, a finding corroborated by a substantial body of prior evidence. A systematic review has demonstrated that prolonged exercise interventions not only confer improvements in physical function and emotional wellbeing but also sustain these benefits throughout extended follow-up periods, suggesting that sustained engagement in physical activity may yield more enduring therapeutic effects (Shu et al., 2014). With respect to the mechanisms underpinning the observed duration-related effects, previous investigations have provided a multifaceted evidence base. Specifically, the induction of structural and functional neuroadaptations may necessitate a minimum threshold of sustained exercise exposure, with interindividual variability and baseline physical fitness constituting critical determinants in establishing the appropriate intervention duration (Xu et al., 2019). A systematic review by El Hayek et al. (2023) noted that only about half of PD physical therapy trials show sustained benefit after regimen completion, supporting the need for interventions longer than 8 weeks.

Our results revealed that interventions with session durations of 60 min or longer produced a larger effect than those of shorter duration. This finding suggests that a minimum threshold of time per session may be necessary to achieve meaningful antidepressant benefits, potentially by allowing sufficient time for neurobiological changes such as endorphin release, neurotrophin upregulation, and sustained engagement of mood-regulating neural circuits (Rodrigues de Paula et al., 2006). The benefits of adequate session duration may also relate to the physiological characteristics of patients with Parkinson’s disease, who often expend greater energy during physical activity compared with healthy individuals (States et al., 2011). Therefore, sessions of appropriate duration may strike a balance between therapeutic efficacy and tolerability.

In terms of session frequency, our findings revealed that interventions delivered less than three times per week showed stronger effects compared with higher frequency. This finding may appear counterintuitive but may be explained by factors related to adherence and tolerability in this patient population. Patients with Parkinson’s disease commonly experience fatigue, motor fluctuations, and reduced energy reserves, which may make high-frequency exercise regimens challenging to sustain (Feng et al., 2020). Programs with moderate frequency may therefore achieve better adherence, resulting in more consistent engagement and ultimately greater symptom improvement. Alternatively, the observed pattern may reflect that studies with lower frequency often employed multicomponent or mind–body interventions, which themselves demonstrated larger effect sizes, introducing potential confounding between frequency and intervention type (Tollár et al., 2018a; Wu et al., 2021). A cross-sectional study by Kinger et al. (2025) found that exercising ≥ 2 days/week was associated with less depression, but fatigue was a major barrier to higher frequency, supporting the feasibility of lower-frequency regimens.

Similarly, interventions with total weekly exercise time of less than 180 min showed superior efficacy compared with those of 180 min or more. This finding aligns with the observation regarding session frequency, suggesting that for patients with Parkinson’s disease, moderate weekly exercise volume may be more feasible and sustainable than higher volumes (Speelman et al., 2011). Excessive weekly exercise time may lead to fatigue and reduced adherence, potentially diminishing antidepressant effects (Dauwan et al., 2021). Therefore, for patients with Parkinson’s disease, achieving an appropriate balance between exercise dose and tolerability may be more important than maximizing volume.

Notably, while our subgroup analyses identified favorable parameters associated with larger effect sizes (e.g., session duration ≥60 min, intervention duration >8 weeks, frequency <3 times per week, and total weekly time <180 min), it is clinically important to recognize that lower doses also produced significant antidepressant effects. Specifically, sessions shorter than 60 min, program duration of 8 weeks or less, frequency of three or more times per week, and total weekly time of 180 min or more all significantly reduced depressive symptoms. This finding aligns with the emerging concept of “exercise snacks” promoted by Trinh et al. (2026), which suggests that even small, accumulated amounts of exercise may be beneficial for individuals with Parkinson’s disease with depressive symptomatology who struggle with adherence. Fatigue is a common and debilitating symptom in this population, and high-volume or high-frequency exercise regimens may exacerbate fatigue, leading to poor adherence and diminished long-term benefits. Therefore, the demonstration that lower-dose regimens are effective provides flexible, patient-centered options that can be tailored to individual energy levels and preferences.

### 4.4 Limitations of the included studies

Several limitations inherent to the included studies warrant consideration. First, comprehensive documentation of exercise intensity was lacking in the majority of trials; most provided only qualitative descriptions (e.g., “moderate intensity”) or omitted intensity data entirely, and even when reported, intensity was often prescribed as a broad range without session-by-session achieved values. This precluded subgroup analysis or dose–response modeling based on intensity, a critical parameter in exercise prescription. Second, medication status—particularly the use of antidepressants or dopaminergic agents that may influence mood outcomes—was inconsistently documented across studies. Although we required that medication dosages be comparable between groups or stable during the intervention when reported, the absence of detailed, longitudinal medication data prevented dose-based subgroup analysis or meta-regression. Third, the included studies varied substantially in setting (hospital-based vs. community-based, inpatient vs. outpatient, individual vs. group format), supervision mode (remote supervision, Parkinson peer-led, highly trained professional-led, or self-managed with or without a care partner), timing of the intervention relative to disease diagnosis (e.g., early-stage vs. advanced), and seasonality of trial conduct—factors that may moderate antidepressant effects but were insufficiently reported to allow further analysis. Furthermore, it should be noted that none of the included RCTs employed a pure high-intensity interval training (HIIT) protocol. Most aerobic exercise interventions consisted of moderate-intensity continuous training. Therefore, the claim that multicomponent exercise yielded “the largest effect size and the most pronounced efficacy” was strictly based on comparisons among the three categories that were actually examined in our subgroup analysis: aerobic exercise, resistance exercise, and multicomponent exercise. HIIT was not part of this comparison, and our findings should not be extrapolated to HIIT protocols.

Additionally, our meta-analytic procedure was not without limitations. Specifically, we did not calculate a kappa statistic for the assessment of methodological quality, nor did we record the exact percentage agreement between the two independent reviewers prior to reaching consensus. Consequently, the results pertaining to methodological quality should be interpreted with appropriate caution.

## 5 Conclusion

This systematic review and meta-analysis demonstrates that exercise significantly alleviates depressive symptoms in people with Parkinson’s disease. For people with mild to moderate Parkinson’s disease with depression, multicomponent exercise appeared to be the most effective intervention type among those examined; however, aerobic and resistance exercise also demonstrated significant benefits. While favorable dosing was observed for sessions of 60 min or longer, program duration exceeding 8 weeks, and a frequency of less than three times per week with total weekly time under 180 min, it is critical to re-emphasize that other exercise regimens were also effective. Specifically, sessions shorter than 60 min, program duration of 8 weeks or less, frequency of three or more times per week, and total weekly time of 180 min or more each produced significant antidepressant effects. These findings support the incorporation of structured exercise programs, including multicomponent, aerobic, and resistance training, into standard care for Parkinson’s disease with depression. Clinicians may consider these evidence-based parameters when prescribing exercise to this patient population, while recognizing that multiple effective dosing options exist.

## Data Availability

The original contributions presented in the study are included in the article/supplementary material, further inquiries can be directed to the corresponding authors.
